# Gastric amyloidosis presenting as acute upper gastrointestinal bleeding: a case report

**DOI:** 10.1186/s12876-021-01882-7

**Published:** 2021-07-29

**Authors:** Rachael Chan, Stephanie Carpentier

**Affiliations:** 1 Department of Medicine, Dalhousie University and Nova Scotia Health, QEII Health Sciences Centre, VG Site, Suite 442 Bethune Building, 1276 South Park Street, Halifax, NS B3H 2Y9 Canada; 2 Division of Gastroenterology, Saint John Regional Hospital, 400 University Avenue, Saint John, NB E2L 4L2 Canada

**Keywords:** Upper GI bleeding, Amyloidosis, Case report

## Abstract

**Background:**

Amyloidosis is characterized by extracellular tissue deposition of fibrils, composed of insoluble low-molecular-weight protein subunits. The type, location, and extent of fibril deposition generates variable clinical manifestations. Gastrointestinal (GI) bleeding due to amyloid deposition is infrequent. Previous literature describes upper GI bleeding (UGIB) in patients with known amyloid disease. Here, we describe a case of recurrent UGIB that ultimately led to a diagnosis of GI amyloidosis and multiple myeloma in a patient with no history of either.

**Case presentation:**

A 76-year-old male presented to the emergency department with frank hematemesis, melena, and a decreased level of consciousness. Management required intensive care unit (ICU) admission with transfusion, intubation, and hemodynamic support. Upper endoscopy revealed gastritis with erosions and nodularity in the gastric cardia and antrum. Hemostasis of a suspected bleeding fundic varix could not be achieved. Subsequently, the patient underwent computerized tomography (CT) angiography and an interventional radiologist completed embolization of the left gastric artery to address potentially life-threatening bleeding. Complications included development of bilateral pleural effusions and subsegmental pulmonary emboli. Pleural fluid was negative for malignancy. He was transferred to a peripheral hospital for continued care and rehabilitation. Unfortunately, he began re-bleeding and was transferred back to our tertiary center, requiring re-admission to the ICU and repeat endoscopy. Repeat biopsy of the gastric cardial nodularity was reported as active chronic gastritis and ulceration. However, based on the unusual endoscopic appearance, clinical suspicion for malignancy remained high. He exhibited symptoms of congestive heart failure following standard resuscitation. Transthoracic echocardiogram (TTE) demonstrated a reduced ejection fraction of 35–40% and a strain pattern with apical sparing. Following discussions between the treating gastroenterologist, consulting cardiologist, and pathologist, Congo Red staining was performed, revealing submucosal amyloid deposits. Hematology was consulted and investigations led to diagnosis of multiple myeloma (MM) and immunoglobulin light-chain (AL) amyloidosis. The patient was treated for MM for four months prior to cessation of therapy due to functional and cognitive decline.

**Conclusions:**

GI amyloidosis can present with various non-specific clinical symptoms and endoscopic findings, rendering diagnosis a challenge. This case illustrates GI amyloidosis as a potential—albeit rare—etiology of UGIB.

## Background

Upper gastrointestinal bleeding (UGIB) is a common cause of emergency department visits and hospital admissions. Peptic ulcer disease, gastritis, and esophagitis have shared risk factors and are the most common causes of UGIB, accounting for 79% of cases [1].

Amyloidosis is characterized by extracellular deposition of fibrils, composed of insoluble low-molecular-weight protein subunits [2]. There are many forms of amyloidosis, classified based on the origin of the deposited protein. Clinical manifestations depend on the type, location, and extent of fibril deposition. Gastrointestinal (GI) amyloidosis can present with non-specific symptoms and endoscopic findings, causing diagnostic difficulty [3]. There have been case reports of UGIB from amyloid deposition in the setting of a known diagnosis of multiple myeloma (MM) [4–6]. We present a case of UGIB, with subsequent investigations leading to the diagnosis of gastric light-chain (AL) amyloidosis and MM.

## Case presentation

A 76-year-old male with coronary artery disease, hypertension, dyslipidemia, and gout presented to hospital with frank hematemesis, melena, and a decreased level of consciousness. He denied any alcohol intake and was a previous smoker. Physical exam was non-contributory other than his decreased level of consciousness. He was admitted to the intensive care unit (ICU), requiring intubation, transfusion, and vasopressor support.

Esophagogastroduodenoscopy (EGD) revealed diffuse gastritis, as well as nodularity and ulceration of the cardia. Hemostasis of what appeared to be a bleeding fundic varix was not performed due to potential diagnostic uncertainty. Subsequent computerized tomography (CT) angiography was completed for clarification, which showed contrast extravasation from the left gastric artery. Given that hemostasis was not achieved on index endoscopy, radiologic evidence of bleeding, and the patient’s critical condition, the treating interventional radiologist performed embolization of the left gastric artery. Imaging did not suggest portal hypertension but showed irregularity along the gastric cardia and fundus. Initial biopsies were reported as chronic gastritis, negative for *H.pylori* or malignancy.

**Fig. 1:**
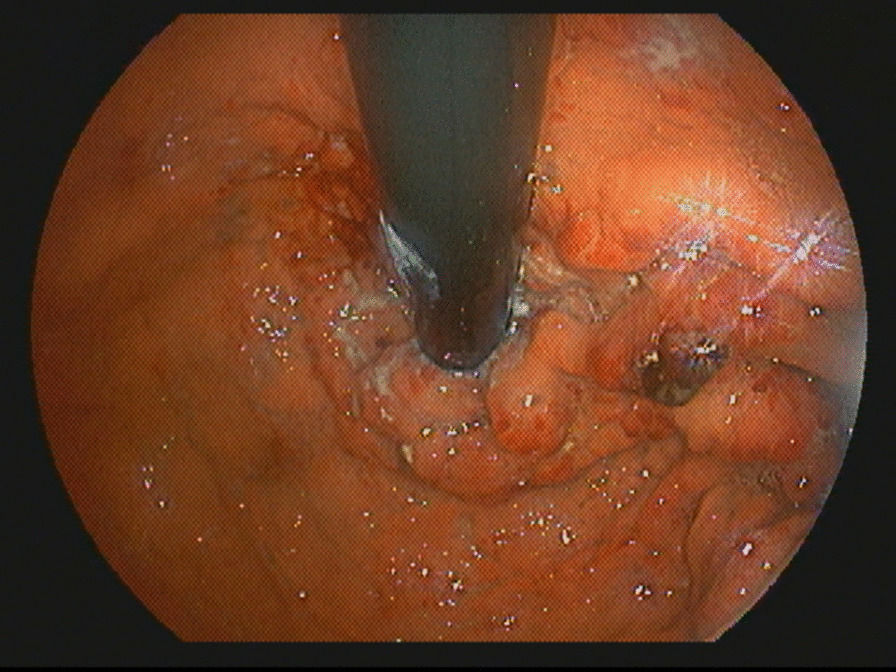
Full view of gastric cardia showing nodularity, edema, and surrounding ulceration

Complications during index admission included development of bilateral subsegmental pulmonary emboli and bilateral pleural effusion. Treatment included therapeutic anticoagulation and bilateral thoracentesis, with pleural fluid analysis consistent with a transudative effusion, negative for malignancy. The patient developed symptoms of significant congestive heart failure following standard resuscitation. Chest radiograph showed vascular congestion. Transthoracic echocardiogram (TTE) showed a strain pattern with apical sparing, moderate left ventricular systolic dysfunction, and an ejection fraction of 35–40%. Repeat endoscopy was arranged to clarify findings and revealed persistent nodularity and ulceration of the cardia (Fig. 1). The patient’s suggestive cardiac manifestations and unexplained appearance on EGD led to case discussion between treating gastroenterologist, consulting cardiologist, and pathologist, prompting staining of gastric biopsies for amyloid. In the interim, the patient was transferred to a peripheral hospital for continued care and rehabilitation prior to results being available.

Unfortunately, the patient developed signs and symptoms of recurrent UGIB, warranting transfer back to our tertiary hospital for re-admission to the ICU. EGD on this occasion revealed a duodenal ulcer with a visible vessel which was treated with 1:10,000 epinephrine injection and cautery. Hemostasis was achieved. It was at this point that prior gastric biopsy results became available. Congo Red staining revealed apple green birefringent material under polarized light, consistent with amyloid deposition.

Subsequent investigations during this re-admission revealed elevated serum kappa light-chains and an elevated kappa/lambda ratio. Serum protein electrophoresis showed an abnormal 3 g/L band of IgG kappa. A diagnosis of multiple myeloma was made. The patient was followed by hematology and actively treated with cyclophosphamide, bortezomib, and dexamethasone (CyBorD) treatment protocol for four months. Despite no recurrence of GI bleeding, the patient experienced progressive weakness, functional and cognitive decline, and was ultimately unable to tolerate further chemotherapy. Subsequently, based on the patient’s goals of care, curative therapy was suspended, comfort care was prioritized, and the patient passed away peacefully.

## Discussion and conclusions

Amyloidosis can be acquired or genetic and is classified based on etiology of the deposited insoluble low-molecular-weight protein subunits. The most common forms of acquired amyloidosis are AL, as in our case; serum amyloid A (AA), which is an acute phase reactant and complication of chronic inflammatory disease; and β-2 microglobulin, which is dialysis related with protein accumulation due to decreased renal clearance. The continuous protein deposition and build-up damages the integrity and function of affected organs.

AL amyloidosis is caused by plasma cell dyscrasia and deposition of immunoglobulin light chains. Determining the epidemiology of AL amyloidosis is difficult due to its rarity. AL amyloidosis is also challenging to diagnose, given its various presenting clinical syndromes. American data reported in 2018 suggests the incidence and prevalence of AL amyloidosis is between 9.7 and 14.0 cases per million person-years and 40.5 cases per million, respectively [7].The majority of patients with AL amyloidosis are over 65-years-old. The most common clinical presentations of AL amyloidosis are nephrotic syndrome and restrictive cardiomyopathy [8]. Tissue biopsy stained with Congo Red showing green birefringence under polarized light is the diagnostic gold standard [2]. The sensitivity and specificity of biopsy location is variable and may involve abdominal fat pad aspiration or biopsy of the organ in which amyloid is suspected, acknowledging increased risks of hemorrhage with the latter. Rectal biopsy is another surrogate site utilized to diagnose systemic amyloidosis with a reported sensitivity of 85% but is no longer recommended as a first-line option for diagnosis due to patient discomfort, risk of complications, and low diagnostic yield in the setting of negative fat pad aspiration [9].

Treatment of AL amyloidosis aims to reduce the production of fibrils to limit deposition–and therefore damage–within amyloidogenic organs. There are many therapeutic regimens used to treat AL amyloidosis, but lack of randomized data comparing efficacy makes decisions surrounding therapy difficult. Since the underlying pathophysiology is plasma cell dyscrasia, management has similarities to that of MM. However, outcomes are better in AL amyloidosis than MM. Treatment options include autologous stem cell transplant (ASCT) and various chemotherapy regimens such as: melphalan and dexamethasone (MDex), cyclophosphamide, thalidomide and dexamethasone (CTDa), and CyBorD [2].

We performed a review of the literature regarding AL amyloidosis in the GI system. PubMed database was used. No date restrictions were used. Only English-language reports were included. Case report, retrospective chart review, and previous systemic reviews that discussed presentation, diagnosis, and management of gastrointestinal amyloidosis were included. There exists no higher-level evidence. Search Terms used: “amyloid”, “AL amyloidosis” “amyloidosis”, “gastrointestinal amyloidosis”, “endoscopy amyloidosis”. 31 results were obtained and formed the basis for this focused review. Articles were further selected based on relevance.

Previous retrospective chart reviews suggest that Gl tract involvement is uncommon in AL amyloidosis, with biopsy proven [10] and clinically apparent [11] disease in 3% and 1% of patients, respectively. The majority of patients with GI amyloid have systemic involvement [10]. There are various GI manifestations of AL amyloidosis, including weight loss, GI bleeding, heartburn, and nausea [10]. Common sites of AL amyloid infiltration include the duodenum, stomach, colorectum, and esophagus [3, 10, 12].With regards to endoscopic abnormalities in those with AL amyloidosis and GI symptoms, 50% present with GI bleeding secondary to ischemia, vascular friability, or mucosal/submucosal lesions [13]. Endoscopic appearances of GI manifestations of AL amyloidosis are highly variable, differ based on anatomic location, and are not specific to amyloidosis subtype [3]. The most common gastric findings include depressed lesions (45%), erythema (32%), edema (29%), mucosal friability (26%), and elevated lesions similar to submucosal tumors (26%); conversely, there may be no gastric findings (45%) [3].

Therapy for GI manifestations of AL amyloidosis are primarily supportive including nutritional supplementation and treatment of diarrhea or obstruction [14].There are no treatment guidelines for endoscopic management of UGIB secondary to AL amyloid disease [9]. Based on a prospective cohort, the prognosis of those with GI manifestations of AL amyloidosis is worse compared to those without GI sequelae, with median survival reported as 7.95 months and 15.84 months, respectively [15].

Recent case reports describe UGIB in patients with known multiple myeloma or monoclonal gammopathy of unknown significance [16, 17]. This case differs because the patient lacked a prior diagnosis and his presentation of recurrent UGIB led to the discovery of his underlying AL amyloidosis. Advanced and irreversible organ dysfunction often precedes the diagnosis of amyloidosis [2]. Therefore, a high index of suspicion and an accessible target organ for diagnostic examination are of utmost importance in expediting treatment and management. Overall, GI amyloidosis presenting as recurrent UGIB is a rare, but known manifestation of AL amyloid disease. This case highlights GI amyloidosis as a differential diagnostic consideration for UGIB and provides an additional example of its endoscopic appearance.

## Data Availability

Not applicable.

## Abbreviations

GI: Gastrointestinal
UGIB: Upper gastrointestinal bleeding
AL: Light chain
ICU: Intensive care unit
EGD: Esophagogastroduodenoscopy
CT: Computerized tomography
CyBorD: Cyclophosphamide, bortezomib, and dexamethasone
AA: Serum amyloid A
MM: Multiple myeloma
ASCT: Autologous stem cell transplant
MDex: Melphalan and dexamethasone
CTDa: Cyclophosphamide, thalidomide and dexamethasone
